# The Effects of Vibegron Add-on Therapy on Alpha 1-Blocker Therapy for Sexual Function and Overactive Bladder Symptoms in Benign Prostatic Hyperplasia: A Prospective, Open-Label Study

**DOI:** 10.3390/jcm13133940

**Published:** 2024-07-05

**Authors:** Kazuki Yanagida, Daisuke Watanabe, Takahiro Yoshida, Akio Mizushima, Tohru Nakagawa

**Affiliations:** 1 Department of Urology, Koto Hospital, Tokyo 136-0072, Japan; yanagidak@med.teikyo-u.ac.jp (K.Y.); t.yoshida@med.teikyo-u.ac.jp (T.Y.); 2 Department of Urology, Teikyo University School of Medicine, Tokyo 173-8606, Japan; nakagawat@med.teikyo-u.ac.jp; 3 Department of Molecular and Cellular Therapeutics, Juntendo University Graduate School of Medicine, Tokyo 113-8421, Japan; akiom@juntendo.ac.jp; 4 Department of Palliative Medicine, Juntendo University Graduate School of Medicine, Tokyo 113-8421, Japan

**Keywords:** benign prostatic hyperplasia, International Index of Erectile Function, male incontinence, overactive bladder symptom, sexual function, vibegron

## Abstract

**Background**: The effect of combining an α_1_-adrenergic receptor blocker (α_1_-blocker) and the β3-adrenoceptor agonist vibegron for treating persistent overactive bladder (OAB) symptoms associated with benign prostatic hyperplasia (BPH) on sexual function remains uncertain. Therefore, we aimed to evaluate the effects of vibegron as an add-on to α_1_-blocker therapy on both OAB and sexual function. **Methods**: Forty-three patients with BPH in whom OAB symptoms were inadequately controlled by α_1_-blocker treatment were included in this prospective open-label study. The OAB Symptom Score (OABSS), International Prostate Symptom Score (IPSS), 15-item International Index of Erectile Function (IIEF-15), and Erection Hardness Score (EHS), as well as the residual urine volume and serum-free testosterone (FT) and C-reactive protein (CRP) levels, were evaluated before and 8 weeks after the daily administration of 50 mg vibegron/α_1_-blocker combination therapy. **Results**: Vibegron/α_1_-blocker combination therapy significantly improved the OABSS (from 6.9 ± 2.6 to 5.1 ± 2.9, *p* < 0.0001) and IIEF intercourse satisfaction domain (from 1.1 ± 2.3 to 1.9 ± 2.6, *p* = 0.02). No significant differences were observed for the IPSS, EHS, total IIEF-15 score, residual urine volume, and serum FT and CRP levels. **Conclusions**: The study findings suggest that vibegron/α_1_-blocker combination therapy improves OAB and sexual satisfaction.

## 1. Introduction

Approximately 50–75% of patients with benign prostatic hyperplasia (BPH) have overactive bladder (OAB) symptoms. Therefore, treating voiding and storage symptoms is important. The α_1_-adrenergic receptor blocker (α_1_-blocker), one of the first-choice drugs for BPH, improves detrusor overactivity and effectively treats OAB symptoms, even when used alone [1,2,3]. However, if OAB symptoms persist after α_1_-blocker therapy, anticholinergic drugs or β3-adrenoceptor agonists are administered concomitantly [1,2,3]. Anticholinergics should be administered while considering the side effects (including dry mouth, blurred vision, and constipation) due to systemic muscarinic receptor blockade. Mirabegron, a β3-adrenoceptor agonist that may prevent the side effects of anticholinergics, has been used widely. However, mirabegron has adverse effects such as tachycardia and blood pressure fluctuations on the cardiovascular system. Therefore, drug interactions, particularly in elderly individuals with multiple comorbidities, need to be considered [4]. In 2018, 50 mg vibegron was approved in Japan as a novel selective β3-adrenoceptor agonist. Vibegron has fewer effects on the cardiovascular system and fewer drug interactions than mirabegron and exhibits no contraindications for coadministration [5].

OAB is associated with an increased risk of erectile dysfunction in men, and the two may share pathophysiological mechanisms [6]. Four pathophysiological mechanisms have been proposed so far: (i) the nitric oxide synthase/nitric oxide theory, (ii) the autonomic hypersensitivity and metabolic syndrome hypothesis, (iii) the Rho kinase activation/endothelin pathway, and (iv) pelvic arteriosclerosis [7,8]. Although phosphodiesterase type 5 inhibitors (PDE5is) are the first-choice drugs for erectile dysfunction, this treatment approach is sometimes ineffective or contraindicated. In recent years, new treatments targeting pathways other than the NO-cGMP pathway by inducing relaxation and suppressing contraction of the corpus cavernosum have been considered for patients in whom PDE5is were ineffective. β3-adrenoceptors are present in the human corpus cavernosum and may play an important role in penile erection [8,9]. Mirabegron, the first β3-adrenoceptor agonist approved for the treatment of OAB, acts simultaneously on the bladder and corpus cavernosum via β3-adrenoceptor activation and could potentially treat erectile dysfunction and OAB [9,10]. The effect of vibegron/α_1_-blocker combination therapy on persistent OAB symptoms associated with BPH and sexual function has not been studied so far.

Therefore, we conducted a prospective, open-label study to assess the effect of vibegron, as an add-on therapy to α_1_-blockers, on both OAB and sexual function.

## 2. Materials and Methods

### 2.1. Statement on Ethics

This study was conducted in accordance with the Declaration of Helsinki, approved by the ethical committee of Koto Hospital (IRB No. 2019126), and registered in the UMIN Clinical Trials Registry (UMIN ID 000039150, https://center6.umin.ac.jp/cgi-open-bin/ctr_e/ctr_view.cgi?recptno=R000044653, accessed on 5 June 2024). Written informed consent was obtained from all the patients.

### 2.2. Study Design and Participants

**Figure 1:**
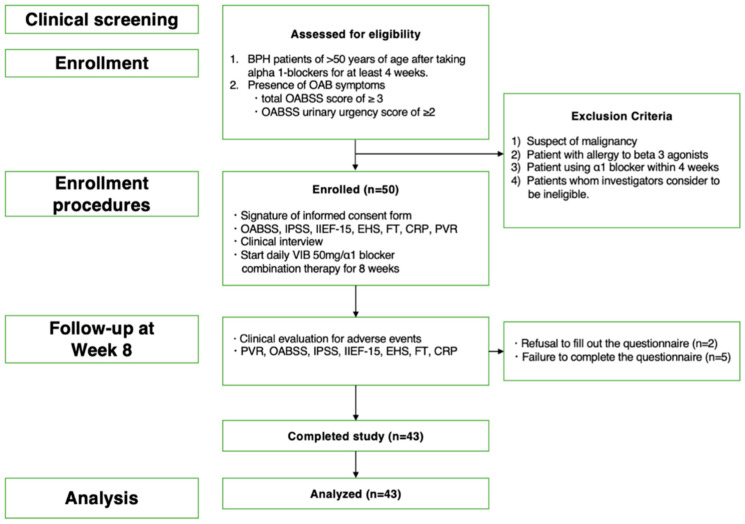
The flowchart shows the inclusion and exclusion criteria, number of patients enrolled, follow-up procedures and endpoints, and number of patients who completed the study and were included in the final analysis.

This prospective, non-blinded study included 50 patients with BPH, aged ≥50 years, with OAB symptoms that were not adequately controlled with α_1_-blockers. The study was conducted between December 2019 and June 2020. The participants included patients with OAB following treatment with an α_1_-blocker for BPH, with a total OAB Symptom Score (OABSS) ≥ 3 and a Q3 score ≥ 2, who consented to participate in this study. Exclusion criteria were suspected malignancy, allergy to the β3-adrenoceptor agonist, the administration of an α_1_-blocker for less than 4 weeks, and being deemed inappropriate by the physician. The OABSS, International Prostate Symptom Score (IPSS), 15-item International Index of Erectile Function (IIEF-15), and Erectile Hardness Score (EHS) measured before daily administration of 50 mg vibegron/α_1_-blocker combination therapy and 8 weeks after administration were assessed along with age, prostate volume, residual urine volume, degree of obesity, and serum-free testosterone (FT) and C-reactive protein (CRP) levels (Figure 1).

### 2.3. Outcome

The primary endpoint was the change in the OABSS 8 weeks after starting vibegron/α_1_-blocker combination therapy. The secondary endpoints were changes in the IIEF-15, EHS, IPSS, FT levels, and high-sensitivity CRP levels 8 weeks after starting vibegron/α_1_-blocker combination therapy.

### 2.4. Statistical Analysis

The expected response rate was set to 30%, based on a threshold response rate of 17.1% in an existing study of mirabegron added to tamsulosin to treat overactive bladders in Asian men [11]. The required sample size of the present single arm study was estimated based on 80% power and an alpha value of 0.1 (one-sided) using the binomial test. Considering 10% were drop-out cases, the sample size was determined to be 50 patients. All clinical data were statistically analyzed using JMP Pro 16 software (SAS Institute, Cary, NC, USA). Paired *t*-tests were applied for clinical indices (continuous values) at baseline and 8 weeks after starting vibegron/α_1_-blocker combination therapy. Statistical significance was set at *p* < 0.05.

## 3. Results

In total, fifty patients were enrolled in the present study; however, seven were excluded from the study, including two who did not wish to take the second questionnaire after 8 weeks of treatment for personal reasons and five whose questionnaires were incomplete. Ultimately, 43 patients were included in the analysis. Adverse events following the administration of vibegron were transient (dry mouth, insomnia, and urinary tract infection in one case each), with no serious adverse events reported during the study period (Figure 1). More than half of the patients (22/43) had hypertension; however, hypertension was not a side effect.

Table 1 shows the clinical characteristics of patients at baseline. The average age was 73.9 ± 4.8 years, body mass index was 23.3 ± 2.9 kg/m^2^, prostate volume was 44.5 ± 21.1 mL, and prostate-specific antigen level was 2.6 ± 3.7 ng/mL (Table 1). Stratification by the type of α_1_-blocker indicated that tamsulosin (0.2 mg/day) was administered to 17 patients, naftopidil (50 or 75 mg/day) to 18 patients, and silodosin (8 mg/day) to 8 patients (Table 1). The cohort included 10 (23.3%) patients with diabetes, 22 (51.2%) with hypertension, 8 (18.6%) with hyperlipidemia, 3 (7.5%) with cardiovascular disease, and none with mental illness; 14 patients (32.6%) were smokers (Table 1).

**Figure 2:**
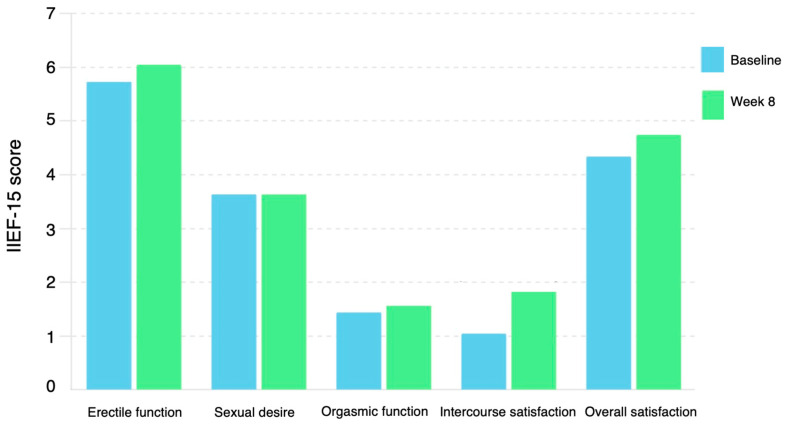
Change in mean IIEF-15 domains from baseline to week 8. IIEF, international index of erectile function.

The 8-week vibegron/α_1_-blocker combination therapy significantly improved the OABSS (from 6.9 ± 2.6 to 5.1 ± 2.9, *p* < 0.0001) after BPH treatment with an α_1_-blocker (Table 2). No significant differences were observed in the IPSS or residual urine volume (Table 2). The 8-week vibegron/α_1_-blocker combination therapy significantly improved the IIEF intercourse satisfaction domain (from 1.1 ± 2.3 to 1.9 ± 2.6, *p* = 0.02) for OAB after BPH treatment with an α_1_-blocker (Table 2) (Figure 2). No significant differences were observed in the EHS, total IIEF-15 score, IIEF erectile function domain, IIEF orgasmic function domain, IIEF sexual desire domain, or IIEF overall satisfaction domain. Moreover, no significant differences were observed in the serum FT and CRP levels.

## 4. Discussion

In this study, we evaluated the effects of vibegron/α_1_-blocker combination therapy on both OAB and sexual function. This combination therapy significantly improved the OABSS and IIEF intercourse satisfaction domains. No significant differences were observed in the IPSS, EHS, total IIEF-15 score, residual urine volume, and serum FT and CRP levels. These results indicate that combining vibegron with an α_1_-blocker may improve OAB and sexual satisfaction.

Although vibegron, a β3-adrenoceptor agonist, is an oral drug that effectively treats OAB symptoms, its effect on sexual function is unknown. Studies have been conducted on the effect on sexual function of mirabegron, another β3-adrenoceptor agonist [12]. A large-scale multinational study that systematically investigated the relationship between lower urinary tract symptoms and sexual dysfunction found that mirabegron improved both erectile function and OAB symptoms in elderly men with OAB and mild to moderate erectile dysfunction [7]. β3-adrenoceptor activation mediates corpus cavernosum relaxation primarily through the cAMP-PKA pathway [9]. Other known mechanisms include the direct activation of K^+^ channels, closure of voltage-gated Ca^2+^ channels, accumulation of cGMP by NO release, and stimulation of the H2S pathway [12]. The authors also described the effect of α_1_-adrenergic receptor (α_1_-AR) blockade on improving erectile function, independent of cAMP accumulation, and reported that mirabegron inhibits smooth muscle contraction induced by phenylephrine (α_1_-AR activation) in human and rat corpus cavernosum [13]. The mechanism of corpus cavernosum relaxation in these mirabegron studies may also apply to vibegron, a β3-adrenoceptor agonist. After 8 weeks of add-on therapy with vibegron, we found no significant improvement in the total IIEF score or erectile function domain and serum FT and CRP levels, which are related to erectile function [14,15]. The low baseline IIEF-EF domain score and EHS and the high number of patients with severe ED may be the reasons underlying the failure of vibegron to contribute to erection function. Although mirabegron has been speculated to likely contribute to erections, no significant results were obtained in the present study [16]. Future studies are warranted when the evaluation is adjusted for confounding factors in patient background. The absence of changes in testosterone levels and systemic inflammation in vibegron combination therapy was not influenced by the mechanism by which sexual satisfaction improved. This result indicates that improved lower urinary tract symptoms could improve sexual satisfaction.

The Boston Area Community Health (BACH) survey among 5503 community residents (aged 30–79 years) in Boston, MA, USA, reported that lower urinary tract symptoms were associated with decreased libido in both men and women [16]. A bivariate analysis that controlled for the effects of diabetes and other comorbidities indicated that low libido in men was associated with depression and nocturia [16]. Furthermore, a survey of Asian men aged > 40 years in a cross-sectional population-representative internet-based self-administered survey conducted in China, Taiwan, and South Korea reported that the severity of lower urinary tract symptoms was negatively associated with libido, intercourse satisfaction, and overall satisfaction [17]. A report investigating the effect of lower urinary tract symptoms on sexual satisfaction in a Brazilian male population found that the presence of lower urinary tract symptoms was significantly associated with sexual satisfaction. Reportedly, among men with lower urinary tract symptoms, the percentage of men complaining of sexual dissatisfaction was significantly higher (13.8%) compared to that of men without lower urinary tract symptoms (4.5%) [18].

Regardless of race, lower urinary tract symptoms, particularly OAB symptoms such as nocturia, negatively impact libido and sexual satisfaction. The significant improvement in the OABSS by vibegron may have positively affected the IIEF intercourse satisfaction domain elevation in our study. A comprehensive assessment of lower urinary tract symptoms, including urinary function and sexual function and satisfaction, in examining lower urinary tract symptoms is important for determining the most appropriate treatment strategy for each patient [18,19]. Our results support that vibegron, a treatment for lower urinary tract symptoms, may also positively affect sexual function and comprehensively improve the quality of life.

This study had certain limitations. First, the study was based on a small sample size and was a single-arm study with no control group as well as a single-center study. Second, three α_1_-blockers may have influenced the study results. Although we aimed to conduct a small initial study of vibegron on OAB and sexual function, future long-term follow-up studies and randomized controlled trials with a large sample size are necessary to further investigate the effects of vibegron on sexual function.

## 5. Conclusions

The β3-adrenoceptor agonist vibegron not only improved male incontinence but also potentially enhanced sexual satisfaction. This finding provides a valuable piece of information for the clinical management of patients with BPH who experience sexual dysfunction.

## Figures and Tables

**Table 1:** Baseline patient characteristics.

Total	No. (%)	43(100)
Age, years	Mean ± SD	73.9 ± 4.8
Body Mass Index, kg/m^2^	Mean ± SD	23.2 ± 2.9
Diabetes mellitus		
Yes	No. (%)	10(23.3)
No	No. (%)	33(76.7)
Controlled hypertension		
Yes	No. (%)	22(51.2)
No	No. (%)	21(48.8)
Hyperlipidemia		
Yes	No. (%)	8(18.6)
No	No. (%)	35(81.4)
Cardiovascular disease		
Yes	No. (%)	3(6.9)
No	No. (%)	40(93.1)
Psychiatric disease		
Yes	No. (%)	0(0)
No	No. (%)	43(100)
History of smoking		
Yes	No. (%)	14(32.6)
No	No. (%)	29(67.4)
Type of α_1_-blocker		
Tamsulosin	No. (%)	17(39.5)
Naftopidil	No. (%)	18(41.9)
Silodosin	No. (%)	8(18.6)
Prostatic volume, mL	Mean ± SD	44.5 ± 21.1
PSA, ng/dL	Mean ± SD	2.6 ± 3.7

PSA, prostate-specific antigen; SD, standard deviation.

**Table 2:** Effect of 8 weeks of vibegron/α_1_-blocker combination therapy.

	Baseline	Week 8	*p* Value
OABSS	6.9 ± 2.6	5.1 ± 2.9	<0.0001
IPSS	16.0 ± 8.1	14.0 ± 8.0	0.0518
IIEF-15 total	16.5 ± 12.5	18.2 ± 14.8	0.3106
Erectile function	5.8 ± 6.2	6.1 ± 7.7	0.7147
Sexual desire	3.7 ± 1.9	3.7 ± 1.9	1
Orgasmic function	1.5 ± 2.7	1.7 ± 2.9	0.6241
Intercourse satisfaction	1.1 ± 2.3	1.9 ± 2.6	0.02
Overall satisfaction	4.4 ± 1.9	4.8 ± 1.7	0.2276
EHS	1.7 ± 1.3	1.7 ± 1.4	0.875
FT, pg/mL	7.2 ± 2.3	6.8 ± 2.9	0.2526
CRP, mg/dL	0.3 ± 0.7	0.2 ± 0.2	0.1517
PVR, mL	66.3 ± 29.0	75.7 ± 55.9	0.3084

Values are presented as mean ± SD. OABSS, overactive bladder symptom score; IPSS, international prostate symptom score; IIEF, international index of erectile function; EHS, erection hardness score; PVR, post-void residual urine; FT, free testosterone; CRP, c-reactive protein.

## Data Availability

Data are contained within the article.
